# Developing a complex intervention to support timely engagement with palliative care for patients with advanced cancer in primary and secondary care in the UK: a study protocol

**DOI:** 10.1136/bmjopen-2018-022835

**Published:** 2018-05-14

**Authors:** Julia Hackett, Hilary Bekker, Michael I Bennett, Paul Carder, Jean Gallagher, Claire Henry, Suzanne Kite, Sally Taylor, Galina Velikova, Lucy Ziegler

**Affiliations:** 1 Academic Unit of Palliative Care, Leeds Institute of Health Sciences, University of Leeds, Leeds, UK; 2 Psychological and Social Medicine, Leeds Institute of Health Sciences, University of Leeds, Leeds, UK; 3 NHS Bradford and Districts CCGs, Bradford, UK; 4 Hospice UK, London, UK; 5 Leeds Teaching Hospitals NHS Trust, St James’s University Hospital, Leeds, UK; 6 Christie Patient Centred Research, The Christie NHS Foundation Trust, Manchester, UK

**Keywords:** palliative care, decision aid, advanced cancer

## Abstract

**Introduction:**

For patients with advanced cancer, timely access to palliative care can improve quality of life and enable patients to participate in decisions about their end-of-life care. However, in a UK population of 2500 patients who died from cancer, one-third did not receive specialist palliative care, and of those who did, the duration of involvement was too short to maximise the benefits. Initiating a conversation about palliative care is challenging for some health professionals and patients often have unmet information needs and misconceptions about palliative care. We will work closely with patients and health professionals to develop a patient decision aid and health professional training module designed to facilitate a timely and informed conversation about palliative care.

**Methods and analysis:**

This study is being conducted over 24 months from November 2017 to October 2019 and follows the UK Medical Research Council framework for developing complex interventions and the International Patient Decision Aids Guideline. The Ottawa Decision Support Framework underpins the study. The Supporting Timely Engagement with Palliative care (STEP) intervention will be developed though an iterative process informed by interviews and focus groups with patients with advanced cancer, oncologists, general practitioners and palliative care doctors. An expert panel will also review each iteration. The expert panel will consist of a patient representative with experience of palliative care, health professionals who are involved in advanced cancer care decision-making, a medical education expert and the National Council for Palliative Care director of transformation. The feasibility and acceptability of the decision aid and doctor training will be tested in oncology and general practice settings.

**Ethics and dissemination:**

Ethical approval for the study has been granted by the Office for Research Ethics Committees Northern Ireland (ORECNI), approval reference 17/NI/0249. Dissemination and knowledge transfer will be conducted via publications, national bodies and networks, and patient and family groups.

**Strengths and limitations of this study**

- To the best of our knowledge, this is the first study to develop a patient decision aid and health professional training module designed to facilitate a timely and informed conversation about referral to palliative care.
- Evidence synthesis and close collaboration with patients and health professionals will enable us to iteratively develop an intervention that overcomes existing barriers to integration of palliative care within cancer services.
- The research will follow the UK MRC framework for developing complex interventions and the International Patient Decision Aids guideline, and be underpinned by the Ottawa Decision Support Framework.
- The research will provide evidence of its acceptability and feasibility to patients and healthcare professionals within routine care in oncology outpatient clinics and general practitioner practices.
- The research will be conducted in one country, the UK. While directly relevant to inform policy and practice in this context, it is possible that patient and health professional experiences of the existing barriers to integration of palliative care within cancer services may vary across countries.

## Introduction

The aim of palliative care is to relieve suffering and improve the quality of life of patients with advanced illnesses. The value of timely integration of palliative care was acknowledged over 15 years ago when WHO incorporated the phrase ‘relief of suffering by means of *early identification* and impeccable assessment’ within its 2002 definition of palliative care. For patients with advanced cancer, several randomised controlled trials^1–5^ have shown that early access to palliative care can improve quality of life, reduce acute hospital admissions, minimise aggressive cancer treatments and enable patients to make choices about their end-of-life care, including exercising the choice to die at home. Earlier integration of palliative care can also improve pain control^6^ and help address inequalities in access to good-quality end-of-life care.^7^ The accumulating evidence to support early referral is beginning to influence policy in the USA^8^; in 2012, the American Society of Clinical Oncology issued guidance to its members recommending ‘palliative care should be considered early in the course of illness for any patient with metastatic cancer and/or high symptom burden’.^7 9^

Previous research suggests that 3–6 months of palliative care is required in order to achieve high-quality end-of-life care.^1–5 8^ However, the current timing of referral to specialist palliative care services in the UK is considerably shorter than this, which is likely to limit the provision of high-quality end-of-life care.^7 10^ Although health professionals in the UK acknowledge the importance of timely referral,^11^ they recognise that in practice, referral does not usually occur until a patient is nearing end of life or experiences an acute episode.^11–13^ Barriers to referral reported by health professionals include attempts to delay the termination of active treatment, belief that they would be abandoning the patient, difficulty initiating the conversation about palliative care and lack of expertise dealing with end-of-life issues. Patient barriers include misconceptions regarding the role of hospices and Macmillan nurses,^14 15^ including assumptions that palliative care was only for patients at the very end of life and a lack of appreciation of the breadth of services provided.^16^ The misconceptions reported by some studies suggest that palliative care should be explained in more detail when it is first introduced.^14^

Tomlinson^16^ identified a lack of written patient information about the role of the specialist palliative care services as a barrier to timely referral. To explore the level of patient information material available locally, we undertook a survey of 12 National Health Service (NHS) Trusts (17 inpatient wards, 12 outpatient wards, 12 day units and 12 palliative care teams) across Yorkshire and Humber in June 2016. Only four of the Trusts surveyed had written information accessible to patients outside of palliative care. This was typically given to patients after they had received a referral to palliative care and focused on practical issues such as contact numbers for palliative care team rather than addressing the prevalent misconceptions about palliative care.^13^ Most of the palliative care teams had information leaflets available, but four specifically said that it was either out of date or that they preferred not to use it. There is an evident lack of understanding of palliative care from the patients’ perspective and a lack of information provision and resources to support decision-making. Given the barriers described above, we explored whether there was existing evidence to support the use of decision aids in this context.

Decision support tools, also known as decision aids, are used in a range of healthcare conditions, and have been defined as an intervention to help patients make informed choices and encourage them to consider their own thoughts and values.^17–21^ Typically, such tools have four components: summarising evidence-informed and best practice options; presenting patients and families with the risks and benefits of the choices available to them^22 23^; checking patients’ understanding of their options; clarify personal values and preferences that influence choices. There is evidence they can reduce patients’ uncertainty, increase patients’ knowledge, help create realistic expectations and increase participation in decision-making.^24^ Although decision aids have been shown to be effective in various healthcare settings,^25–28^ our systematic searches of national and international decision aid registries and databases identified only four decision aids, which related broadly to end-of-life issues such as resuscitation decisions and decisions relating to preferred place of death. We have been unable to identify any existing decision aids to support decisions about palliative care referral. There is a clear lack of information provision in oncology settings and a surprising lack of existing decision aids to help patients make informed decisions about palliative care referral. The latter may be partly explained by decision aids being reliant on the provision of evidence-based information to support the options under consideration. It is only since 2011 that reliable evidence has become available to demonstrate the benefit of palliative care to patients with advanced cancer.^1–5 8^

The proposed project addresses an issue that is timely in terms of government policy,^29–31^ is a national clinical priority and has the potential to be used in other healthcare contexts across the NHS to engage non-cancer populations with palliative care. By drawing on stakeholder consultation, guided by the UK Medical Research Council framework for developing complex interventions and the International Patient Decision Aids Standards (IPDAS), and underpinned by the Ottawa Decision Support Framework (ODSF), this study aims to develop and implement an intervention to help patients with advanced cancer and their clinicians make timely informed decisions about palliative care.

## Methods and analysis

This research protocol details the process of development and validation of a decision aid for patients with advanced cancer and related doctor training for clinicians. The study will be conducted over 24 months, from November 2017 to October 2019.

### Patient and public involvement

Patients were involved in setting the research question and in the design of the study. Patients will be asked to advise on interpretation of results. The results of the research will be disseminated to the patient community through patient forums.

### Recruitment

Patients with advanced cancer will be recruited through oncology outpatient clinics and a local hospice via health professionals. Patients will be approached in person and given an information sheet. If they are interested in taking part, they will be contacted by a member of the research team and given the opportunity to ask any questions. Health professionals will be recruited through members of the research team, facilitated by clinical end-of-life care leads within Leeds Cancer Centre and the primary care setting. The member of the research team will introduce the study and provide them with an information sheet. Patients and health professionals will be recruited in the same way for both interviews and focus groups. All participants will be asked to give written consent prior to participation in the study.

### Intervention development and implementation processes

The intervention development will consist of eight steps and the intervention implementation will consist of two steps, outlined (table 1) and detailed below.

**Table 1:** STEP intervention development and implementation process

Step	Objective	Framework	Method
1.1	Convene an expert panel	UKMRC/IPDAS	Expert consensus (face-to-face meetings)
1.2	Needs assessment (completed)	UKMRC/IPDAS	12 Patient and 12 clinician interviews
1.3	Review of existing resources (completed)	UKMRC/IPDAS	Literature review and environmental scan of existing information resources Regional survey of 20 hospitals (26 inpatient/outpatient oncology departments, 12 day units and 12 palliative care teams)
1.4	Evidence: collate the clinical evidence for treatment options (completed)	IPDAS/UKMRC	Time4PallCare Project; retrospective cohort study of 6080 cancer deaths
1.5	Map existing patients’ pathways	UKMRC	(1) Interviews with patients’ oncologists, oncology nurse specialists and general practitioners (2) Retrospective population cohort study
2.1	Develop the prototype intervention and (alpha testing I)	UKMRC/IPDAS	Draft–review–revise iterative process by the research team and design experts
2.2	Review by expert panel, focus groups (alpha testing II)	UKMRC/IPDAS	(1) Expert panel consensus—meeting and emails (2) Focus groups
2.3	Develop the STEP training module for clinicians		(1) Ottawa training module and workshop (2) Development of STEP clinician handbook—expert consensus involving research team, decision support experts and medical educator
3.1	Implementation study (beta testing)	IPDAS	(1) Pilot STEP with patients and their clinicians (2) Patient and clinician questionnaires (3) Individual interviews with patients and clinicians
3.2	Implementation toolkit		(1) Develop toolkit (2) Embed toolkit within SystmOne (3) Create STEP website

IPDAS, International Patient Decision Aids Standards; UKMRC, UK Medical Research Council.

## 1.1 Expert panel consultation process

Step 1.1 will be to convene an expert panel, using the recruitment process described above, to guide the development of STEP, including content format and feasibility testing. They will assess the STEP intervention based on (1) members’ experience as patients with cancer who have made a decision about referral to palliative care, (2) healthcare professionals who are involved with patients in making such decisions and (3) key opinion leaders/policy-makers who are involved in implementing health services for patients with advanced cancer. They will be independent of the research team and project steering committee and will engage with the research team through face-to-face meetings and where necessary via Skype and email correspondence.

### 1.5 Map existing patient pathways

Step 1.5 is the mapping of existing patient with advanced cancer clinical pathways to identify decision points and decision-makers within the pathway and understand how transition between and integration of the pathways currently occur. Interviews will be conducted with 16 patients (eight prior to receiving a referral to palliative care, eight following a referral to palliative care) and eight health professionals. Participants will be recruited using the recruitment processes described above. Interviews will explore perspectives of journey from diagnosis, with particular focus on determining the potential decision points and understanding the role of the patient and others in the decision-making process. Health professional interviews will also explore the extent to which palliative care provision is being delivered by non-palliative care specialists and the rationale for this.

We will use an existing dataset containing linked data relating to 2479 patients with cancer from the cancer registry and the electronic medical record systems used within primary care (SystmOne) and within Leeds Cancer Centre (Patient Pathway Manager). From this linked dataset, we will map patient pathways through primary and secondary care from diagnosis to death. We will explore how the pathways differ in relation to patient, disease and various service level characteristics, what treatment they received, and the number and nature of hospital admissions. This way will enable us to identify potential decision points and key decision-makers and crucially will enable us to navigate transitions between primary care, oncology and palliative care.

### 2.1 Develop the prototype intervention and alpha testing (I)

In step 2.1, a prototype will be developed through synthesis of evidence and expert consensus based on the IPDAS guideline and the ODSF. IPDAS will guide the process for synthesising the research evidence (from steps 1.2 to 1.5) and structuring the STEP intervention.

### 2.2 Review by expert panel and alpha testing (II)

In step 2.2 the decision aid will be refined by patients, health professionals, and an expert panel. This will be done via four focus groups: two with patients (a mix of patients who have received palliative care and those who have not) and two with health professionals (oncologists, general practitioners (GPs) and oncology nurse specialists). Focus groups will explore the comprehensibility and usability of the decision aid. Data will be analysed thematically and the decision aid will be iteratively modified as a result. The expert panel will then meet following these revisions and further iterations will be undertaken until the expert panel reach consensus regarding the content and format of STEP.

### 2.3 Develop the STEP training module for clinicians

Step 2.3 consists of developing and piloting the STEP training module, skills-building workshop and the handbook. Health professionals (four GPs, four oncologists, four oncology nurse specialists) will be recruited to the implementation study and complete both the online tutorial and the skills-building workshop before using the intervention in clinical consultations. The skills-building workshop will take place within Leeds Cancer Centre or the GP practice during protected learning time and will last approximately 90 min. Feedback on the skills development workshop and the online training will be collected at the end of the workshop using a questionnaire.

### 3.1 Implementation study

In step 3.1, we will assess the acceptability and validity of STEP in consultations and its impact on patient care. Health professionals who participated in the training will be asked to use the STEP intervention within their clinical practice. Usage will be explored using interviews, questionnaires (Ottawa Acceptability Tool, Decision Support Analysis Tool and Decision Conflict Scale) and clinical feedback forms. They will each identify patients with advanced cancer who have the potential to benefit from palliative care but have not received a palliative care referral. We will aim to generate a patient population of 36 (3 patients per health professional). If the patient agrees to participate, the clinician will use the STEP intervention in the consultation. Following the consultation, each of the 36 patients will be sent follow-up questionnaires and 12 patients will also be invited to take part in a follow-up interview. Health professionals will be given a structured feedback form on which they will be asked to reflect on the use of the STEP intervention after each consultation in which it is used. They will be invited to participate in a semistructured interview.

### 3.2 Implementation toolkit

In step 3.2, an implementation toolkit will be developed to support the implementation of STEP in oncology departments and General Practice settings across the NHS. The toolkit will provide the necessary information and resources to implement the decision aid and clinical training and will also provide clinically feasible resources to evaluate its impact including The Sure Test. To support sustainability and continued evaluation, the toolkit will be embedded within the central user areas of the two electronic health record systems used in primary care in Yorkshire (EMIS and SystmOne). Any updates or modifications will be therefore be incorporated within routine NHS IT maintenance programmes.

We will also create a STEP website where the implementation toolkit will be available to health professionals who wish to adopt it within their practice. Users will be asked to complete a registration form so we can monitor where the decision aid is being used and we will request feedback on the implementation process within practice settings.

## Ethics and dissemination

Ethical approval for the study has been granted by the Office for Research Ethics Committees Northern Ireland, approval reference 17/NI/0249. Recruitment materials, including information sheets and consent forms, were also approved. Management of participant information will be collected and processed in accordance with the Data Protection Act 1998, that is, all personal information will be de-identified and kept in password-protected electronic files. Information will be destroyed after 5 years.

Research dissemination and knowledge translation will be conducted in three ways. We will build on working relationships with national bodies relevant to end-of-life care and broader health policy in order to shape policy and clinical recommendations for wider dissemination nationally. We will engage with palliative care experts, representatives from hospice networks, and palliative care patient and family groups to inform them of progress and emerging research findings. We will also link with national bodies to ensure our project is aligned with their priorities and channels are available for national dissemination of our work. We will also disseminate findings via traditional academic routes of conferences and peer-reviewed publications.

## Supplementary Material

Reviewer comments

Author's manuscript
