# Supplementary material for: Developing a complex intervention to support timely engagement with palliative care for patients with advanced cancer in primary and secondary care in the UK: a study protocol
Source: BMJ Open. 2018 May 14;8(5):e022835. doi: 10.1136/bmjopen-2018-022835 (PMC5961581; doi:10.1136/bmjopen-2018-022835)
Supplement: Author's manuscript [file bmjopen-2018-022835.draft_revisions.pdf]

BMJ Open is committed to open peer review. As part of this commitment we make the peer review history of every article we publish publicly available.

When an article is published we post the peer reviewers' comments and the authors' responses online. We also post the versions of the paper that were used during peer review. These are the versions that the peer review comments apply to.

The versions of the paper that follow are the versions that were submitted during the peer review process. They are not the versions of record or the final published versions. They should not be cited or distributed as the published version of this manuscript.

BMJ Open is an open access journal and the full, final, typeset and author-corrected version of record of the manuscript is available on our site with no access controls, subscription charges or pay-per-view fees (http://bmjopen.bmj.com).

If you have any questions on BMJ Open's open peer review process please email info.bmjopen@bmj.com

BMJ Open

Developing a complex intervention to support timely engagement with palliative care for patients with advanced cancer in primary and secondary care settings in the UK: A study protocol.

Journal:	*BMJ Open*
Manuscript ID	bmjopen-2018-022835
Article Type:	Protocol
Date Submitted by the Author:	08-Mar-2018

Complete List of Authors:	Hackett, Julia; University of Leeds Leeds Institute of Health Sciences, Academic Unit of Palliative Care<br>Bekker, Hilary; University of Leeds, Leeds Institute of Health Sciences, Psychological & Social Medicine<br>Bennett, Michael; University of Leeds, Leeds Institute of Health Sciences<br>Carder, Paul ; NHS Bradford and Districts CCGs<br>Gallagher, Jean; University of Leeds Leeds Institute of Health Sciences, Academic Unit of Palliative Care<br>Henry, Claire; Hospice UK, 34-44 Britannia Street<br>Kite, Suzanne; Leeds Teaching Hospitals NHS Trust, St James's University Hospital, Beckett Street,<br>Taylor, Sally; The Christie NHS Foundation Trust, Christie Patient Centred Research<br>Velikova, Galina; University of Leeds<br>Ziegler, Lucy; University of Leeds Leeds Institute of Health Sciences, Academic Unit of Palliative Care

Keywords:	Adult palliative care < PALLIATIVE CARE, ONCOLOGY, decision aid, advanced cancer

SCHOLARONE™
Manuscripts

Developing a complex intervention to support timely engagement with palliative care for patients with advanced cancer in primary and secondary care settings in the UK: A study protocol

Hackett J[1]*, Bekker H[2], Bennett MI[1], Carder P[3], Gallagher J[1], Henry C[4], Kite S[5], Taylor S[6], Velikova G[6], Ziegler L[1].

Corresponding author:

Julia Hackett. Academic Unit of Palliative Care, Leeds Institute of Health Sciences, University of Leeds, Worsley Building, Clarendon Way, Leeds, UK.

Email: j.e.hackett@leeds.ac.uk Phone: +44 (0)113 343 0857

Author institution address:

[1] Academic Unit of Palliative Care, Leeds Institute of Health Sciences, University of Leeds, Worsley Building, Clarendon Way, Leeds, UK.

[2] Psychological & Social Medicine, Leeds Institute of Health Sciences, University of Leeds, Worsley Building, Clarendon Way, Leeds, UK.

[3] NHS Bradford Districts CCG, Douglas Mill, Bowling Old Lane, Bradford, West Yorkshire, UK.

[4] Hospice UK, 34-44 Britannia Street, London, UK.

[5] Leeds Teaching Hospitals NHS Trust, St James's University Hospital, Beckett Street, Leeds, West Yorkshire, UK.

[6] The Christie NHS Foundation Trust, Wilmslow Road, Manchester, UK.

Word count: 2965

Keywords: palliative care, advanced cancer, decision aid

For peer review only

Abstract

Introduction

For advanced cancer patients, timely access to palliative care can improve quality of life and enable patients to participate in decisions about their end-of-life care. However in a UK population of 2,500 patients who died from cancer one third did not receive specialist palliative care and of those who did, the duration of involvement was too short to maximise the benefits. Initiating a conversation about palliative care is challenging for some health professionals and patients often have unmet information needs and misconceptions about palliative care. We will work closely with patients and health professionals to develop a patient decision aid and health professional training module designed to facilitate a timely and informed conversation about palliative care.

Methods and Analysis

This study is being conducted over 24 months from November 2017 to October 2019 and follows the UK Medical Research Council framework for developing complex interventions and the International Patient Decision Aids Guideline. The Ottawa Decision Support Framework underpins the study. The STEP intervention will be developed though an iterative process informed by interviews and focus groups with patients with advanced cancer, oncologists, general practitioners and palliative care doctors. An expert panel will also review each iteration. The expert panel will consist of a patient representative with experience of palliative care, health professionals who are involved in advanced cancer care decision-making, a medical education expert and the National Council for Palliative Care director of transformation. The feasibility and acceptability of the decision aid and doctor training will be tested in oncology and general practice settings.

Ethics and Dissemination

Ethical approval for the study has been granted by the Office for Research Ethics Committees Northern Ireland (ORECNI), approval reference 17/NI/0249. Dissemination and knowledge transfer will be conducted via publications, national bodies and networks, and patient and family groups.

Strengths and limitations of this study

- To the best of our knowledge, this is the first study to develop a patient decision aid and health professional training module designed to facilitate a timely and informed conversation about referral to palliative care.

For peer review only

- Evidence synthesis and close collaboration with patients and health professionals will enable us to iteratively develop an intervention which overcomes existing barriers to integration of palliative care within cancer services.
- The research will follow the UK MRC framework for developing complex interventions and the International Patient Decision Aids guideline, and be underpinned by the Ottawa Decision Support Framework.
- The research will provide evidence of its acceptability and feasibility to patients and healthcare professionals within routine care in oncology outpatient clinics and GP practices.
- The research will be conducted in one country, the UK. Whilst directly relevant to inform policy and practice in this context, it is possible that patient and health professional experiences of the existing barriers to integration of palliative care within cancer services, may vary across countries.

Introduction

The aim of palliative care is to relieve suffering and improve the quality of life of patients with advanced illnesses. The value of timely integration of palliative care was acknowledged over 15 years ago when the World Health Organisation incorporated the phrase 'relief of suffering by means of *early identification* and impeccable assessment' within its 2002 definition of palliative care. For patients with advanced cancer, several randomised controlled trials [1-5] have shown that early access to palliative care can improve quality of life, reduce acute hospital admissions, minimise aggressive cancer treatments and enable patients to make choices about their end-of-life care, including exercising the choice to die at home. Earlier integration of palliative care can also improve pain control [6] and help address inequalities in access to good quality end of life care [7]. The accumulating evidence to support early referral is beginning to influence policy in the US [8], in 2012the American Society of Clinical Oncology issued guidance to its members recommending 'palliative care should be considered early in the course of illness for any patient with metastatic cancer and/or high symptom burden' [9, 10].

Previous research suggests that 3-6 months of palliative care is required in order to achieve high quality end-of-life care [1-5, 8]. However, the current timing of referral to specialist palliative care services in the United Kingdom is considerably shorter than this, which is likely to limit the provision of high quality end-of-life care [7, 11]. Although health professionals in the UK acknowledge the importance of timely referral [12] they recognise that in practice, referral does not usually occur

For peer review only

until a patient is nearing end-of-life or experiences an acute episode [12-14]. Barriers to referral reported by health professionals include attempts to delay the termination of active treatment, belief that they would be abandoning the patient, difficulty initiating the conversation about palliative care, and lack of expertise dealing with end-of-life issues. Patient barriers include misconceptions regarding the role of hospices and Macmillan nurses [15, 16], including assumptions that palliative care was only for patients at the very end-of-life and a lack of appreciation of the breadth of services provided [17]. The misconceptions reported by some studies suggest that palliative care should be explained in more detail when it is first introduced [15].

Tomlinson [17] identified a lack of written patient information about the role of the specialist palliative care services as a barrier to timely referral. To explore the level of patient information material available locally, we undertook a survey of 12 NHS Trusts (17 inpatient wards, 12 outpatient wards, 12 day units and 12 palliative care teams) across Yorkshire and Humber in June 2016. Only four of the Trusts surveyed had written information accessible to patients outside of palliative care. This was typically given to patients after they had received a referral to palliative care and focussed on practical issues such as contact numbers for palliative care team rather than addressing the prevalent misconceptions about palliative care [14]. Most of the palliative care teams had information leaflets available, but four specifically said that it was either out of date, or that they preferred not to use it. There is an evident lack of understanding of palliative care from the patients' perspective and a lack of information provision and resources to support decision making. Given the barriers described above, we explored whether there was existing evidence to support the use of decision aids in this context.

Decision support tools, also known as decision aids, are used in a range of healthcare conditions, and have been defined as an intervention to help patients make informed choices and encourage them to consider their own thoughts and values [18-22]. Typically such tools have four components: summarising evidence-informed and best practice options; presenting patients and families with the risks and benefits of the choices available to them[23, 24]; checking patients' understanding of their options; and clarify personal values and preferences that influence choices. There is evidence they can reduce patients' uncertainty; increase patients' knowledge, help create realistic expectations and increase participation in decision making [25]. Although decision aids have been shown to be effective in various healthcare settings [26-29] our systematic searches of national and international decision aid registries and databases identified only four decision aids which related broadly to end-of-life issues such as resuscitation decisions and decisions relating to preferred place of death. We have been unable to identify any existing decision aids to support decisions about palliative care

For peer review only - http://bmjopen.bmj.com/site/about/guidelines.xhtml

referral. There is a clear lack of information provision in oncology settings and a surprising lack of existing decision aids to help patients make informed decisions about palliative care referral. The latter may be partly explained by decision aids being reliant on the provision of evidence based information to support the options under consideration. It is only since 2011 that reliable evidence has become available to demonstrate the benefit of palliative care to patients with advanced cancer [1-5, 8].

The proposed project addresses an issue which is timely in terms of government policy [30-32], is a national clinical priority, and has the potential to be used in other healthcare contexts across the NHS to engage non-cancer populations with palliative care. By drawing on stakeholder consultation, guided by the UK Medical Research Council (UKMRC) framework for developing complex interventions and the International Patient Decision Aids Standards (IPDAS), and underpinned by the Ottawa Decision Support Framework (ODSF), this study aims to develop and implement an intervention to help patients with advanced cancer and their clinicians make timely informed decisions about palliative care.

Methods and analysis

This research protocol details the process of development and validation of a decision aid for patients with advanced cancer and related doctor training for clinicians. The study will be conducted over 24 months, from November 2017 to October 2019.

Recruitment

Patients with advanced cancer will be recruited through oncology outpatient clinics and a local hospice via health professionals. Patients will be approached in person, and given an information sheet. If they are interested in taking part they will be contacted by a member of the research team and given the opportunity to ask any questions. Health professionals will be recruited through members of the research team, facilitated by clinical end-of-life care leads within Leeds Cancer centre and the primary care setting. The member of the research team will introduce the study and provide them with an information sheet. Patients and health professionals will be recruited in the same way for both interviews and focus groups. All participants will be asked to give written consent prior to participation in the study.

Intervention development and implementation processes

The intervention development will consist of 8 steps and the intervention implementation will consist of two steps, outlined (Table 1) and detailed below.

For peer review only

Table 1: STEP intervention development and implementation process

Step	Objective	Framework	Method
1.1	Convene an expert panel	UKMRC/IPDAS	Expert consensus (face-to-face meetings)
1.2	Needs assessment **(Completed)**	UKMRC/IPDAS	12 Patient and 12 Clinician interviews
1.3	Review of existing resources **(Completed)**	UKMRC/IPDAS	Literature review and Environmental scan of existing information resources. Regional survey of 20 hospitals [26 inpatient/outpatient oncology departments, 12 day units and 12 palliative care teams]
1.4	Evidence: Collate the clinical evidence for treatment options **(Completed)**	IPDAS/UKMRC	Time4PallCare Project; retrospective cohort study of 6,080 cancer deaths
1.5	Map existing patients pathways	UKMRC	i) Interviews with patients oncologists, oncology nurse specialists, and GPs. ii)Retrospective population cohort study
2.1	Develop the prototype intervention and (Alpha testing I)	UKMRC/IPDAS	Draft-review-revise iterative process by the research team and design experts
2.2	Review by expert panel, focus groups (Alpha testing II)	UKMRC/IPDAS	i) Expert panel consensus – meeting and emails ii) Focus groups
2.3	Develop the STEP training module for clinicians		i) Ottowa training module and workshop ii) Development of STEP clinician handbook - expert consensus involving research team,

For peer review only

			decision support experts and medical educator
3.1	Implementation study (Beta Testing)	IPDAS	i) Pilot STEP with patients and their clinicians.<br>ii)Patients and clinician questionnaires<br>iii) Individual interviews with patients and clinicians
3.2	Implementation toolkit		i) Develop toolkit<br>ii) Embed toolkit within SystmOne<br>iii) Create STEP website

1.1 Expert panel consultation process

Step 1.1 will be to convene an expert panel, using the recruitment process described above, to guide the development of STEP, including content format and feasibility testing. They will assess the STEP-intervention based on: (1) member's experience as cancer patients who have made a decision about referral to palliative care; (2) healthcare professionals who are involved with patients in making such decisions; and (3) key opinion leaders/policy makers who are involved in implementing health services for patients with advanced cancer. They will be independent of the research team and project steering committee and will engage with the research team through face to face meetings and where necessary via Skype and email correspondence.

1.5 Map existing patient pathways

Step 1.5 is the mapping of existing advanced cancer patient clinical pathways to identify decision points and decision makers within the pathway and understand how transition between and integration of the pathways currently occur. Interviews will be conducted with 16 patients (8 prior to receiving a referral to palliative care, 8 following a referral to palliative care) and 8 health professionals. Participants will be recruited using the recruitment processes described above. Interviews will explore perspectives of journey from diagnosis, with particular focus on determining the potential decision points and understanding the role of the patient and others in the decision making process. Health professional interviews will also explore the extent to which palliative care provision is being delivered by non-palliative care specialists and the rationale for this.

For peer review only

We will use an existing dataset containing linked data relating to 2479 cancer patients from the cancer registry and the electronic medical record systems used within primary care (SystMOne) and within Leeds Cancer Centre (Patient Pathway Manager). From this linked dataset we will map patient pathways through primary and secondary care from diagnosis to death. We will explore how the pathways differ in relation to patient, disease, and various service level characteristics, what treatment they received, and the number and nature of hospital admissions. This way will enable us to identify potential decision points and key decision makers and crucially will enable us to navigate transitions between primary care, oncology and palliative care.

2.1 Develop the prototype intervention and Alpha testing (i)

In step 2.1 a prototype will be developed through synthesis of evidence and expert consensus based on the IPDAS guideline and the ODSF. IPDAS will guide the process for synthesising the research evidence (from steps 1.2-1.5) and structuring the STEP intervention.

2.2 Review by expert panel and Alpha testing (ii)

In step 2.2 the decision aid will be refined by patients, health professionals, and an expert panel. This will be done via four focus groups: two with patients (a mix of patients who have received palliative care and those who have not) and two with health professionals (oncologists GPs and oncology nurse specialists). Focus groups will explore the comprehensibility and usability of the decision aid. Data will be analysed thematically and the decision aid will be iteratively modified as a result. The expert panel will then meet following these revisions and further iterations will be undertaken until the expert panel reach consensus regarding the content and format of STEP.

2.3 Develop the STEP training module for clinicians

Step 2.3 consists of developing and piloting the STEP training module, skills-building workshop, and the handbook. Health professionals (4 GPs, 4 oncologists, 4 oncology nurse specialists) will be recruited to the implementation study, and complete both the online tutorial and the skills building workshop before using the intervention in clinical consultations. The skills building workshop will take place within Leeds Cancer Centre or the GP practice during protected learning time and will last approximately 90 minutes. Feedback on the skills development workshop and the online training will be collected at the end of the workshop using a questionnaire.

3.1 Implementation study

In step 3.1 we will assess the acceptability and validity of STEP in consultations and its impact on patient care. Health professionals who participated in the training will be asked to use the STEP

For peer review only - http://bmjopen.bmj.com/site/about/guidelines.xhtml

intervention within their clinical practice. Usage will be explored using interviews, questionnaires (Ottowa Acceptability Tool, Decision Support Analysis Tool and Decision Conflict Scale) and clinical feedback forms. They will each identify patients with advanced cancer who have the potential to benefit from palliative care but have not received a palliative care referral. We will aim to generate a patient population of 36 (3 patients per health professional). If the patient agrees to participate the clinician will use the STEP intervention in the consultation. Following the consultation each of the 36 patients will be sent follow up questionnaires and 12 patients will also be invited to take part in a follow up interview. Health professionals will be given a structured feedback form on which they will be asked to reflect on the use of the STEP intervention after each consultation in which it is used. They will be invited to participate in a semi-structured interview.

3.2 Implementation toolkit

In step 3.2 an implementation toolkit will be developed to support the implementation of STEP in oncology departments and General Practice settings across the NHS. The toolkit will provide the necessary information and resources to implement the decision aid and clinical training and will also provide clinically feasible resources to evaluate its impact including The Sure Test. To support sustainability and continued evaluation the toolkit will be embedded within the central user areas of the two electronic health record systems used in primary care in Yorkshire (EMIS and SystmOne). Any updates or modifications will be therefore be incorporated within routine NHS IT maintenance programmes.

We will also create a STEP website where the implementation toolkit will be available to health professionals who wish to adopt it within their practice. Users will be asked to complete a registration form so we can monitor where the decision aid is being used and we will request feedback on the implementation process within practice settings.

Ethics and dissemination

Ethical approval for the study has been granted by the Office for Research Ethics Committees Northern Ireland, approval reference 17/NI/0249. Recruitment materials, including information sheets and consent forms, were also approved. Management of participant information will be collected and processed in accordance with the Data Protection Act 1998, that is, all personal information will be de-identified and kept in password protected electronic files. Information will be destroyed after five years.

For peer review only

Research dissemination and knowledge translation will be conducted in three ways. We will build on working relationships with national bodies relevant to end-of-life care and broader health policy in order to shape policy and clinical recommendations for wider dissemination nationally. We will engage with palliative care experts, representatives from hospice networks, palliative care patient and family groups to inform them of progress and emerging research findings. We will also link with national bodies to ensure our project is aligned with their priorities and channels are available for national dissemination of our work. We will also disseminate findings via traditional academic routes of conferences and peer-reviewed publications.

Author contributions

JH drafted the initial manuscript. All authors reviewed the manuscript prior to submission. All authors were involved in the critical revision of the article for important intellectual content and provided final approval of its content.

Funding statement

This article presents independent research funded by the NIHR Research for Patient Benefit Scheme (PB-PG-0416-20004). The views expressed are those of the authors and not necessarily those of the NHS, the NIHR or the Department of Health. The sponsor and funders had no involvement in the study design; collection, management, analysis, and interpretation of data; writing of the report; and the decision to submit the report for publication.

Competing interests

We have read and understood BMJ policy on declaration of interests and declare no interests.

Data Sharing

No additional data available.

Transparency statement

The lead author affirms that the manuscript is an honest, accurate, and transparent account of the study being reported; that no important aspects of the study have been omitted; and that any discrepancies are disclosed.

References

For peer review only

1. Bakitas M, Lyons KD, Hegel MT, Balan S, Barnett KN, Brokaw FC, Byock IR, Hull JG, Li Z, McKinstry E, et al: **The project ENABLE II randomized controlled trial to improve palliative care for rural patients with advanced cancer: baseline findings, methodological challenges, and solutions.** *Palliat Support Care* 2009, **7:**75-86.
2. Rabow MW, Dibble SL, Pantilat SZ, McPhee SJ: **The comprehensive care team: a controlled trial of outpatient palliative medicine consultation.** *Arch Intern Med* 2004, **164:**83-91.
3. Temel JS, Greer JA, Muzikansky A, Gallagher ER, Admane S, Jackson VA, Dahlin CM, Blinderman CD, Jacobsen J, Pirl WF, et al: **Early palliative care for patients with metastatic non-small-cell lung cancer.** *N Engl J Med* 2010, **363:**733-742.
4. Zimmermann C, Swami N, Krzyzanowska M, Hannon B, Leighl N, Oza A, Moore M, Rydall A, Rodin G, Tannock I, et al: **Early palliative care for patients with advanced cancer: a cluster-randomised controlled trial.** *Lancet* 2014, **383:**1721-1730.
5. Brumley R, Enguidanos S, Jamison P, Seitz R, Morgenstern N, Saito S, McIlwane J, Hillary K, Gonzalez J: **Increased satisfaction with care and lower costs: results of a randomized trial of in-home palliative care.** *J Am Geriatr Soc* 2007, **55:**993-1000.
6. Ziegler L, Mulvey M, Blenkinsopp A, Petty D, Bennett MI: **Opioid prescribing for patients with cancer in the last year of life: a longitudinal population cohort study.** *Pain* 2016, **157:**2445-2451.
7. Bennett MI, Ziegler L, Allsop M, Daniel S, Hurlow A: **What determines duration of palliative care before death for patients with advanced disease? A retrospective cohort study of community and hospital palliative care provision in a large UK city.** *BMJ Open* 2016, **6**.
8. Mazanec P, Prince-Paul M: **Integrating palliative care into active cancer treatment.** *Semin Oncol Nurs* 2014, **30:**203-211.
9. Smith TJ, Temin S, Alesi ER, Abernethy AP, Balboni TA, Basch EM, Ferrell BR, Loscalzo M, Meier DE, Paice JA, et al: **American Society of Clinical Oncology provisional clinical opinion: the integration of palliative care into standard oncology care.** *J Clin Oncol* 2012, **30:**880-887.
10. Bennett MI, Ziegler L, Allsop M, Daniel S, Hurlow A: **What determines duration of palliative care before death for patients with advanced disease? A retrospective cohort study of community and hospital palliative care provision in a large UK city.** *BMJ Open* 2016, **6**.
11. Ziegler LE, Craigs CL, West RM, Carder P, Hurlow A, Millares-Martin P, Hall G, Bennett MI: **Is palliative care support associated with better quality outcomes at end of life for patients with advanced cancer? A longitudinal population cohort study.** *BMJ Open* (Under Review).
12. Johnson C, Girgis A, Paul C, Currow DC, Adams J, Aranda S: **Australian palliative care providers' perceptions and experiences of the barriers and facilitators to palliative care provision.** *Support Care Cancer* 2011, **19:**343-351.
13. Charalambous H, Pallis A, Hasan B, O'Brien M: **Attitudes and referral patterns of lung cancer specialists in Europe to Specialized Palliative Care (SPC) and the practice of Early Palliative Care (EPC).** *BMC Palliat Care* 2014, **13:**59.
14. Taylor S, Ziegler L: **Patients' undertsanding of palliative care, is the information good enough? A literature review and audit of information provision.** *Cancer Nursing Practice* In press.
15. Randall TC, Wearn AM: **Receiving bad news: patients with haematological cancer reflect upon their experience.** *Palliat Med* 2005, **19:**594-601.
16. Hackett J, Godfrey M, Bennett MI: **The process of managing advanced cancer pain toward the end of life: the patient and carer perspective.** *Palliative Medicine* 2016, **30:**711-719.
17. Tomlinson K, Barker S, Soden K: **What are cancer patients' experiences and preferences for the provision of written information in the palliative care setting? A focus group study.** *Palliat Med* 2012, **26:**760-765.

For peer review only

For peer review only - http://bmjopen.bmj.com/site/about/guidelines.xhtml

18. Stacey D, Bennett CL, Barry MJ, Col NF, Eden KB, Holmes-Rovner M, Llewellyn-Thomas H, Lyddiatt A, Legare F, Thomson R: **Decision aids for people facing health treatment or screening decisions.** *Cochrane Database Syst Rev* 2011, **10**.

19. Bekker H, Winterbottom A, Butow P, Dillard A, Feldman-Stewart D, Fowler J, al. e: **Do personal stories make patient decision aids more effective: a critical review of evidence and theory?** *BMC Med Inform Decis Mak* 2013, **13 (Suppl 2):**S2-9.

20. Bekker H: **The loss of reason in patient decision aid research: do checklists affect the validity of informed choice interventions?** *Patient Educ Couns* 2010, **78:**357-364.

21. Bekker H, Thornton JG, Airey CM, Connelly JB, Hewison J, Lilleyman J, al. e: **Informed decision making: an annotated bibliography and systematic review.** *Health Technol Assess* 1999, **3:**1-56.

22. Winterbottom A, Gavaruzzi T, Mooney A, Wilkie M, Davies S, Crane D, Tupling K, Baxter PD, Meads DM, Mathers N, Bekker H: **Patient Acceptability of the Yorkshire Dialysis Decision Aid (YoDDA) Booklet: A Prospective Non-Randomized Comparison Study Across 6 Predialysis Services.** *Perit Dial Int* 2016, **36:**374-381.

23. Elwyn G, Frosch D, Thomson R, Joseph-Williams N, Lloyd A, Kinnersley P, Cording E, Tomson D, Dodd C, Rollnick S, et al: **Shared decision making: A model for clinical practice.** *Journal of General Internal Medicine* 2012, **27:**1361-1367.

24. Legare F, Politi MC, VDrolet R, Desroches S, Stacey D, Bekker H, team. tS-C: **Training health professionals in shared decision-making: an international environmental scan.** *Patient Educ Couns* 2012, **88:**159-169

25. O'Connor A: **Using patient decision aids to promote evidence-based decision making.** *ACP J Club* 2001, **135:**A11-12.

26. Stacey D, Bennett CL, Barry MJ, Col NF, Eden KB, Holmes-Rovner M, Llewellyn-Thomas H, Lyddiatt A, Legare F, Thomson R: **Decision aids for people facing health treatment or screening decisions.** *Cochrane Database Syst Rev* 2011:CD001431.

27. O'Connor AM, Rostom A, Fiset V, Tetroe J, Entwistle V, Llewellyn-Thomas H, Holmes-Rovner M, Barry M, Jones J: **Decision aids for patients facing health treatment or screening decisions: systematic review.** *BMJ* 1999, **319:**731-734.

28. Molenaar S, Sprangers MA, Postma-Schuit FC, Rutgers EJ, Noorlander J, Hendriks J, de Haes HC: **Feasibility and effects of decision aids.** *Med Decis Making* 2000, **20:**112-127.

29. Abhyankar P, Velikova G, Summers B, Bekker HL: **Identifying components in consent information needed to support informed decision making about trial particiaption: An interview study with women managing cancer.** *Social Science & Medicine* 2016, **161:**83-91.

30. The association for Palliative Medicine of Great Britain and Ireland Cnipcrg, Marie Curie Cancer Care, National Council for palliative Care and Palliative care Section of the Royal Society of Medicine,: **Commissioning Guidance for Specialist Palliative Care: Helping to deliver commissioning objectives.** London; 2012.

31. The Department of Health: **End of Life Care Strategy – promoting high quality care for all adults at the end of life.** 2008.

32. National Institute for Health and Care Excellence: **Quality Standard for end of life care for adults.** 2013.

For peer review only

BMJ Open

Developing a complex intervention to support timely engagement with palliative care for patients with advanced cancer in primary and secondary care in the UK: A study protocol

Journal:	*BMJ Open*
Manuscript ID	bmjopen-2018-022835.R1
Article Type:	Protocol
Date Submitted by the Author:	24-Apr-2018
Complete List of Authors:	Hackett, Julia; University of Leeds Leeds Institute of Health Sciences, Academic Unit of Palliative Care<br>Bekker, Hilary; University of Leeds, Leeds Institute of Health Sciences, Psychological & Social Medicine<br>Bennett, Michael; University of Leeds, Leeds Institute of Health Sciences, Academic Unit of Palliative Care<br>Carder, Paul ; NHS Bradford and Districts CCGs<br>Gallagher, Jean; University of Leeds Leeds Institute of Health Sciences, Academic Unit of Palliative Care<br>Henry, Claire; Hospice UK, 34-44 Britannia Street<br>Kite, Suzanne; Leeds Teaching Hospitals NHS Trust, St James's University Hospital, Beckett Street,<br>Taylor, Sally; The Christie NHS Foundation Trust, Christie Patient Centred Research<br>Velikova, Galina; University of Leeds<br>Ziegler, Lucy; University of Leeds Leeds Institute of Health Sciences, Academic Unit of Palliative Care
**Primary Subject Heading**:	Palliative care
Secondary Subject Heading:	Oncology, Qualitative research
Keywords:	Adult palliative care < PALLIATIVE CARE, ONCOLOGY, decision aid, advanced cancer

SCHOLARONE™
Manuscripts

Developing a complex intervention to support timely engagement with palliative care for patients with advanced cancer in primary and secondary care in the UK: A study protocol

Hackett J[1]*, Bekker H[2], Bennett MI[1], Carder P[3], Gallagher J[1], Henry C[4], Kite S[5], Taylor S[6], Velikova G[6], Ziegler L[1].

Corresponding author:

Julia Hackett. Academic Unit of Palliative Care, Leeds Institute of Health Sciences, University of Leeds, Worsley Building, Clarendon Way, Leeds, UK.

Email: j.e.hackett@leeds.ac.uk Phone: +44 (0)113 343 0857

Author institution address:

[1] Academic Unit of Palliative Care, Leeds Institute of Health Sciences, University of Leeds, Worsley Building, Clarendon Way, Leeds, UK.

[2] Psychological & Social Medicine, Leeds Institute of Health Sciences, University of Leeds, Worsley Building, Clarendon Way, Leeds, UK.

[3] NHS Bradford Districts CCG, Douglas Mill, Bowling Old Lane, Bradford, West Yorkshire, UK.

[4] Hospice UK, 34-44 Britannia Street, London, UK.

[5] Leeds Teaching Hospitals NHS Trust, St James's University Hospital, Beckett Street, Leeds, West Yorkshire, UK.

[6] The Christie NHS Foundation Trust, Wilmslow Road, Manchester, UK.

Word count: 2965

Keywords: palliative care, advanced cancer, decision aid

For peer review only

Abstract

Introduction

For advanced cancer patients, timely access to palliative care can improve quality of life and enable patients to participate in decisions about their end-of-life care. However in a UK population of 2,500 patients who died from cancer one third did not receive specialist palliative care and of those who did, the duration of involvement was too short to maximise the benefits. Initiating a conversation about palliative care is challenging for some health professionals and patients often have unmet information needs and misconceptions about palliative care. We will work closely with patients and health professionals to develop a patient decision aid and health professional training module designed to facilitate a timely and informed conversation about palliative care.

Methods and Analysis

This study is being conducted over 24 months from November 2017 to October 2019 and follows the UK Medical Research Council framework for developing complex interventions and the International Patient Decision Aids Guideline. The Ottawa Decision Support Framework underpins the study. The STEP intervention will be developed though an iterative process informed by interviews and focus groups with patients with advanced cancer, oncologists, general practitioners and palliative care doctors. An expert panel will also review each iteration. The expert panel will consist of a patient representative with experience of palliative care, health professionals who are involved in advanced cancer care decision-making, a medical education expert and the National Council for Palliative Care director of transformation. The feasibility and acceptability of the decision aid and doctor training will be tested in oncology and general practice settings.

Ethics and Dissemination

Ethical approval for the study has been granted by the Office for Research Ethics Committees Northern Ireland (ORECNI), approval reference 17/NI/0249. Dissemination and knowledge transfer will be conducted via publications, national bodies and networks, and patient and family groups.

Strengths and limitations of this study

- To the best of our knowledge, this is the first study to develop a patient decision aid and health professional training module designed to facilitate a timely and informed conversation about referral to palliative care.

For peer review only

- Evidence synthesis and close collaboration with patients and health professionals will enable us to iteratively develop an intervention which overcomes existing barriers to integration of palliative care within cancer services.
- The research will follow the UK MRC framework for developing complex interventions and the International Patient Decision Aids guideline, and be underpinned by the Ottawa Decision Support Framework.
- The research will provide evidence of its acceptability and feasibility to patients and healthcare professionals within routine care in oncology outpatient clinics and GP practices.
- The research will be conducted in one country, the UK. Whilst directly relevant to inform policy and practice in this context, it is possible that patient and health professional experiences of the existing barriers to integration of palliative care within cancer services, may vary across countries.

Introduction

The aim of palliative care is to relieve suffering and improve the quality of life of patients with advanced illnesses. The value of timely integration of palliative care was acknowledged over 15 years ago when the World Health Organisation incorporated the phrase 'relief of suffering by means of *early identification* and impeccable assessment' within its 2002 definition of palliative care. For patients with advanced cancer, several randomised controlled trials [1-5] have shown that early access to palliative care can improve quality of life, reduce acute hospital admissions, minimise aggressive cancer treatments and enable patients to make choices about their end-of-life care, including exercising the choice to die at home. Earlier integration of palliative care can also improve pain control [6] and help address inequalities in access to good quality end of life care [7]. The accumulating evidence to support early referral is beginning to influence policy in the US [8], in 2012the American Society of Clinical Oncology issued guidance to its members recommending 'palliative care should be considered early in the course of illness for any patient with metastatic cancer and/or high symptom burden' [9, 10].

Previous research suggests that 3-6 months of palliative care is required in order to achieve high quality end-of-life care [1-5, 8]. However, the current timing of referral to specialist palliative care services in the United Kingdom is considerably shorter than this, which is likely to limit the provision of high quality end-of-life care [7, 11]. Although health professionals in the UK acknowledge the importance of timely referral [12] they recognise that in practice, referral does not usually occur

For peer review only

until a patient is nearing end-of-life or experiences an acute episode [12-14]. Barriers to referral reported by health professionals include attempts to delay the termination of active treatment, belief that they would be abandoning the patient, difficulty initiating the conversation about palliative care, and lack of expertise dealing with end-of-life issues. Patient barriers include misconceptions regarding the role of hospices and Macmillan nurses [15, 16], including assumptions that palliative care was only for patients at the very end-of-life and a lack of appreciation of the breadth of services provided [17]. The misconceptions reported by some studies suggest that palliative care should be explained in more detail when it is first introduced [15].

Tomlinson [17] identified a lack of written patient information about the role of the specialist palliative care services as a barrier to timely referral. To explore the level of patient information material available locally, we undertook a survey of 12 NHS Trusts (17 inpatient wards, 12 outpatient wards, 12 day units and 12 palliative care teams) across Yorkshire and Humber in June 2016. Only four of the Trusts surveyed had written information accessible to patients outside of palliative care. This was typically given to patients after they had received a referral to palliative care and focussed on practical issues such as contact numbers for palliative care team rather than addressing the prevalent misconceptions about palliative care [14]. Most of the palliative care teams had information leaflets available, but four specifically said that it was either out of date, or that they preferred not to use it. There is an evident lack of understanding of palliative care from the patients' perspective and a lack of information provision and resources to support decision making. Given the barriers described above, we explored whether there was existing evidence to support the use of decision aids in this context.

Decision support tools, also known as decision aids, are used in a range of healthcare conditions, and have been defined as an intervention to help patients make informed choices and encourage them to consider their own thoughts and values [18-22]. Typically such tools have four components: summarising evidence-informed and best practice options; presenting patients and families with the risks and benefits of the choices available to them[23, 24]; checking patients' understanding of their options; and clarify personal values and preferences that influence choices. There is evidence they can reduce patients' uncertainty; increase patients' knowledge, help create realistic expectations and increase participation in decision making [25]. Although decision aids have been shown to be effective in various healthcare settings [26-29] our systematic searches of national and international decision aid registries and databases identified only four decision aids which related broadly to end-of-life issues such as resuscitation decisions and decisions relating to preferred place of death. We have been unable to identify any existing decision aids to support decisions about palliative care

For peer review only

referral. There is a clear lack of information provision in oncology settings and a surprising lack of existing decision aids to help patients make informed decisions about palliative care referral. The latter may be partly explained by decision aids being reliant on the provision of evidence based information to support the options under consideration. It is only since 2011 that reliable evidence has become available to demonstrate the benefit of palliative care to patients with advanced cancer [1-5, 8].

The proposed project addresses an issue which is timely in terms of government policy [30-32], is a national clinical priority, and has the potential to be used in other healthcare contexts across the NHS to engage non-cancer populations with palliative care. By drawing on stakeholder consultation, guided by the UK Medical Research Council (UKMRC) framework for developing complex interventions and the International Patient Decision Aids Standards (IPDAS), and underpinned by the Ottawa Decision Support Framework (ODSF), this study aims to develop and implement an intervention to help patients with advanced cancer and their clinicians make timely informed decisions about palliative care.

Methods and analysis

This research protocol details the process of development and validation of a decision aid for patients with advanced cancer and related doctor training for clinicians. The study will be conducted over 24 months, from November 2017 to October 2019.

Patient and public involvement

Patients were involved in setting the research question and in the design of the study. Patients will be asked to advise on interpretation of results. The results of the research will be disseminated to the patient community through patient forums.

Recruitment

Patients with advanced cancer will be recruited through oncology outpatient clinics and a local hospice via health professionals. Patients will be approached in person, and given an information sheet. If they are interested in taking part they will be contacted by a member of the research team and given the opportunity to ask any questions. Health professionals will be recruited through members of the research team, facilitated by clinical end-of-life care leads within Leeds Cancer centre and the primary care setting. The member of the research team will introduce the study and provide them with an information sheet. Patients and health professionals will be recruited in the

For peer review only

same way for both interviews and focus groups. All participants will be asked to give written consent prior to participation in the study.

Intervention development and implementation processes

The intervention development will consist of 8 steps and the intervention implementation will consist of two steps, outlined (Table 1) and detailed below.

Table 1: STEP intervention development and implementation process

Step	Objective	Framework	Method
1.1	Convene an expert panel	UKMRC/IPDAS	Expert consensus (face-to-face meetings)
1.2	Needs assessment **(Completed)**	UKMRC/IPDAS	12 Patient and 12 Clinician interviews
1.3	Review of existing resources **(Completed)**	UKMRC/IPDAS	Literature review and Environmental scan of existing information resources. Regional survey of 20 hospitals [26 inpatient/outpatient oncology departments, 12 day units and 12 palliative care teams]
1.4	Evidence: Collate the clinical evidence for treatment options **(Completed)**	IPDAS/UKMRC	Time4PallCare Project; retrospective cohort study of 6,080 cancer deaths
1.5	Map existing patients pathways	UKMRC	i) Interviews with patients oncologists, oncology nurse specialists, and GPs. ii)Retrospective population cohort study
2.1	Develop the prototype intervention and (Alpha testing I)	UKMRC/IPDAS	Draft-review-revise iterative process by the research team and design experts
2.2	Review by expert panel,	UKMRC/IPDAS	i) Expert panel consensus – meeting

For peer review only

	focus groups (Alpha testing II)		and emails<br>ii) Focus groups
2.3	Develop the STEP training module for clinicians		i) Ottowa training module and workshop<br>ii) Development of STEP clinician handbook - expert consensus involving research team, decision support experts and medical educator
3.1	Implementation study (Beta Testing)	IPDAS	i) Pilot STEP with patients and their clinicians.<br>ii)Patients and clinician questionnaires<br>iii) Individual interviews with patients and clinicians
3.2	Implementation toolkit		i) Develop toolkit<br>ii) Embed toolkit within SystmOne<br>iii) Create STEP website

For peer review only

1.1 Expert panel consultation process

Step 1.1 will be to convene an expert panel, using the recruitment process described above, to guide the development of STEP, including content format and feasibility testing. They will assess the STEP-intervention based on: (1) member's experience as cancer patients who have made a decision about referral to palliative care; (2) healthcare professionals who are involved with patients in making such decisions; and (3) key opinion leaders/policy makers who are involved in implementing health services for patients with advanced cancer. They will be independent of the research team and project steering committee and will engage with the research team through face to face meetings and where necessary via Skype and email correspondence.

1.5 Map existing patient pathways

Step 1.5 is the mapping of existing advanced cancer patient clinical pathways to identify decision points and decision makers within the pathway and understand how transition between and integration of the pathways currently occur. Interviews will be conducted with 16 patients (8 prior

For peer review only - http://bmjopen.bmj.com/site/about/guidelines.xhtml

to receiving a referral to palliative care, 8 following a referral to palliative care) and 8 health professionals. Participants will be recruited using the recruitment processes described above. Interviews will explore perspectives of journey from diagnosis, with particular focus on determining the potential decision points and understanding the role of the patient and others in the decision making process. Health professional interviews will also explore the extent to which palliative care provision is being delivered by non-palliative care specialists and the rationale for this.

We will use an existing dataset containing linked data relating to 2479 cancer patients from the cancer registry and the electronic medical record systems used within primary care (SystMOne) and within Leeds Cancer Centre (Patient Pathway Manager). From this linked dataset we will map patient pathways through primary and secondary care from diagnosis to death. We will explore how the pathways differ in relation to patient, disease, and various service level characteristics, what treatment they received, and the number and nature of hospital admissions. This way will enable us to identify potential decision points and key decision makers and crucially will enable us to navigate transitions between primary care, oncology and palliative care.

2.1 Develop the prototype intervention and Alpha testing (i)

In step 2.1 a prototype will be developed through synthesis of evidence and expert consensus based on the IPDAS guideline and the ODSF. IPDAS will guide the process for synthesising the research evidence (from steps 1.2-1.5) and structuring the STEP intervention.

2.2 Review by expert panel and Alpha testing (ii)

In step 2.2 the decision aid will be refined by patients, health professionals, and an expert panel. This will be done via four focus groups: two with patients (a mix of patients who have received palliative care and those who have not) and two with health professionals (oncologists GPs and oncology nurse specialists). Focus groups will explore the comprehensibility and usability of the decision aid. Data will be analysed thematically and the decision aid will be iteratively modified as a result. The expert panel will then meet following these revisions and further iterations will be undertaken until the expert panel reach consensus regarding the content and format of STEP.

2.3 Develop the STEP training module for clinicians

Step 2.3 consists of developing and piloting the STEP training module, skills-building workshop, and the handbook. Health professionals (4 GPs, 4 oncologists, 4 oncology nurse specialists) will be recruited to the implementation study, and complete both the online tutorial and the skills building workshop before using the intervention in clinical consultations. The skills building workshop will

take place within Leeds Cancer Centre or the GP practice during protected learning time and will last approximately 90 minutes. Feedback on the skills development workshop and the online training will be collected at the end of the workshop using a questionnaire.

3.1 Implementation study

In step 3.1 we will assess the acceptability and validity of STEP in consultations and its impact on patient care. Health professionals who participated in the training will be asked to use the STEP intervention within their clinical practice. Usage will be explored using interviews, questionnaires (Ottowa Acceptability Tool, Decision Support Analysis Tool and Decision Conflict Scale) and clinical feedback forms. They will each identify patients with advanced cancer who have the potential to benefit from palliative care but have not received a palliative care referral. We will aim to generate a patient population of 36 (3 patients per health professional). If the patient agrees to participate the clinician will use the STEP intervention in the consultation. Following the consultation each of the 36 patients will be sent follow up questionnaires and 12 patients will also be invited to take part in a follow up interview. Health professionals will be given a structured feedback form on which they will be asked to reflect on the use of the STEP intervention after each consultation in which it is used. They will be invited to participate in a semi-structured interview.

3.2 Implementation toolkit

In step 3.2 an implementation toolkit will be developed to support the implementation of STEP in oncology departments and General Practice settings across the NHS. The toolkit will provide the necessary information and resources to implement the decision aid and clinical training and will also provide clinically feasible resources to evaluate its impact including The Sure Test. To support sustainability and continued evaluation the toolkit will be embedded within the central user areas of the two electronic health record systems used in primary care in Yorkshire (EMIS and SystmOne). Any updates or modifications will be therefore be incorporated within routine NHS IT maintenance programmes.

We will also create a STEP website where the implementation toolkit will be available to health professionals who wish to adopt it within their practice. Users will be asked to complete a registration form so we can monitor where the decision aid is being used and we will request feedback on the implementation process within practice settings.

Ethics and dissemination

For peer review only

Ethical approval for the study has been granted by the Office for Research Ethics Committees Northern Ireland, approval reference 17/NI/0249. Recruitment materials, including information sheets and consent forms, were also approved. Management of participant information will be collected and processed in accordance with the Data Protection Act 1998, that is, all personal information will be de-identified and kept in password protected electronic files. Information will be destroyed after five years.

Research dissemination and knowledge translation will be conducted in three ways. We will build on working relationships with national bodies relevant to end-of-life care and broader health policy in order to shape policy and clinical recommendations for wider dissemination nationally. We will engage with palliative care experts, representatives from hospice networks, palliative care patient and family groups to inform them of progress and emerging research findings. We will also link with national bodies to ensure our project is aligned with their priorities and channels are available for national dissemination of our work. We will also disseminate findings via traditional academic routes of conferences and peer-reviewed publications.

Author contributions

JH drafted the initial manuscript. JH, LZ, and HB were involved in the planning of the manuscript. All authors reviewed the manuscript prior to submission. All authors were involved in the critical revision of the article for important intellectual content and provided final approval of its content. The team comprises of the primary investigator (LZ) and coinvestigators (HB, MIB, PC, JG, CH, SK, ST, GV). All coinvestigators were involved in the conception and design of the study. We would like to thank the patient advisers for their input and advice in setting the research question and in the design of the study.

Funding statement

This article presents independent research funded by the NIHR Research for Patient Benefit Scheme (PB-PG-0416-20004). The views expressed are those of the authors and not necessarily those of the NHS, the NIHR or the Department of Health. The sponsor and funders had no involvement in the study design; collection, management, analysis, and interpretation of data; writing of the report; and the decision to submit the report for publication.

Competing interests

We have read and understood BMJ policy on declaration of interests and declare no interests.

Data Sharing

For peer review only

No additional data available.

Transparency statement

The lead author affirms that the manuscript is an honest, accurate, and transparent account of the study being reported; that no important aspects of the study have been omitted; and that any discrepancies are disclosed.

References

1. Bakitas M, Lyons KD, Hegel MT, Balan S, Barnett KN, Brokaw FC, Byock IR, Hull JG, Li Z, McKinstry E, et al: **The project ENABLE II randomized controlled trial to improve palliative care for rural patients with advanced cancer: baseline findings, methodological challenges, and solutions.** *Palliat Support Care* 2009, **7:**75-86.
2. Rabow MW, Dibble SL, Pantilat SZ, McPhee SJ: **The comprehensive care team: a controlled trial of outpatient palliative medicine consultation.** *Arch Intern Med* 2004, **164:**83-91.
3. Temel JS, Greer JA, Muzikansky A, Gallagher ER, Admane S, Jackson VA, Dahlin CM, Blinderman CD, Jacobsen J, Pirl WF, et al: **Early palliative care for patients with metastatic non-small-cell lung cancer.** *N Engl J Med* 2010, **363:**733-742.
4. Zimmermann C, Swami N, Krzyzanowska M, Hannon B, Leighl N, Oza A, Moore M, Rydall A, Rodin G, Tannock I, et al: **Early palliative care for patients with advanced cancer: a cluster-randomised controlled trial.** *Lancet* 2014, **383:**1721-1730.
5. Brumley R, Enguidanos S, Jamison P, Seitz R, Morgenstern N, Saito S, McIlwane J, Hillary K, Gonzalez J: **Increased satisfaction with care and lower costs: results of a randomized trial of in-home palliative care.** *J Am Geriatr Soc* 2007, **55:**993-1000.
6. Ziegler L, Mulvey M, Blenkinsopp A, Petty D, Bennett MI: **Opioid prescribing for patients with cancer in the last year of life: a longitudinal population cohort study.** *Pain* 2016, **157:**2445-2451.
7. Bennett MI, Ziegler L, Allsop M, Daniel S, Hurlow A: **What determines duration of palliative care before death for patients with advanced disease? A retrospective cohort study of community and hospital palliative care provision in a large UK city.** *BMJ Open* 2016, **6**.
8. Mazanec P, Prince-Paul M: **Integrating palliative care into active cancer treatment.** *Semin Oncol Nurs* 2014, **30:**203-211.
9. Smith TJ, Temin S, Alesi ER, Abernethy AP, Balboni TA, Basch EM, Ferrell BR, Loscalzo M, Meier DE, Paice JA, et al: **American Society of Clinical Oncology provisional clinical opinion: the integration of palliative care into standard oncology care.** *J Clin Oncol* 2012, **30:**880-887.
10. Bennett MI, Ziegler L, Allsop M, Daniel S, Hurlow A: **What determines duration of palliative care before death for patients with advanced disease? A retrospective cohort study of community and hospital palliative care provision in a large UK city.** *BMJ Open* 2016, **6**.
11. Ziegler LE, Craigs CL, West RM, Carder P, Hurlow A, Millares-Martin P, Hall G, Bennett MI: **Is palliative care support associated with better quality outcomes at end of life for patients with advanced cancer? A longitudinal population cohort study.** *BMJ Open* (Under Review).
12. Johnson C, Girgis A, Paul C, Currow DC, Adams J, Aranda S: **Australian palliative care providers' perceptions and experiences of the barriers and facilitators to palliative care provision.** *Support Care Cancer* 2011, **19:**343-351.

For peer review only

For peer review only - http://bmjopen.bmj.com/site/about/guidelines.xhtml

13. Charalambous H, Pallis A, Hasan B, O'Brien M: **Attitudes and referral patterns of lung cancer specialists in Europe to Specialized Palliative Care (SPC) and the practice of Early Palliative Care (EPC).** *BMC Palliat Care* 2014, **13:**59.
14. Taylor S, Ziegler L: **Patients' undertsanding of palliative care, is the information good enough? A literature review and audit of information provision.** *Cancer Nursing Practice* In press.
15. Randall TC, Wearn AM: **Receiving bad news: patients with haematological cancer reflect upon their experience.** *Palliat Med* 2005, **19:**594-601.
16. Hackett J, Godfrey M, Bennett MI: **The process of managing advanced cancer pain toward the end of life: the patient and carer perspective.** *Palliative Medicine* 2016, **30:**711-719.
17. Tomlinson K, Barker S, Soden K: **What are cancer patients' experiences and preferences for the provision of written information in the palliative care setting? A focus group study.** *Palliat Med* 2012, **26:**760-765.
18. Stacey D, Bennett CL, Barry MJ, Col NF, Eden KB, Holmes-Rovner M, Llewellyn-Thomas H, Lyddiatt A, Legare F, Thomson R: **Decision aids for people facing health treatment or screening decisions.** *Cochrane Database Syst Rev* 2011, **10**.
19. Bekker H, Winterbottom A, Butow P, Dillard A, Feldman-Stewart D, Fowler J, al. e: **Do personal stories make patient decision aids more effective: a critical review of evidence and theory?** *BMC Med Inform Decis Mak* 2013, **13 (Suppl 2):**S2-9.
20. Bekker H: **The loss of reason in patient decision aid research: do checklists affect the validity of informed choice interventions?** *Patient Educ Couns* 2010, **78:**357-364.
21. Bekker H, Thornton JG, Airey CM, Connelly JB, Hewison J, Lilleyman J, al. e: **Informed decision making: an annotated bibliography and systematic review.** *Health Technol Assess* 1999, **3:**1-56.
22. Winterbottom A, Gavaruzzi T, Mooney A, Wilkie M, Davies S, Crane D, Tupling K, Baxter PD, Meads DM, Mathers N, Bekker H: **Patient Acceptability of the Yorkshire Dialysis Decision Aid (YoDDA) Booklet: A Prospective Non-Randomized Comparison Study Across 6 Predialysis Services.** *Perit Dial Int* 2016, **36:**374-381.
23. Elwyn G, Frosch D, Thomson R, Joseph-Williams N, Lloyd A, Kinnersley P, Cording E, Tomson D, Dodd C, Rollnick S, et al: **Shared decision making: A model for clinical practice.** *Journal of General Internal Medicine* 2012, **27:**1361-1367.
24. Legare F, Politi MC, VDrolet R, Desroches S, Stacey D, Bekker H, team. tS-C: **Training health professionals in shared decision-making: an international environmental scan.** *Patient Educ Couns* 2012, **88:**159-169

25. O'Connor A: **Using patient decision aids to promote evidence-based decision making.** *ACP J Club* 2001, **135:**A11-12.
26. Stacey D, Bennett CL, Barry MJ, Col NF, Eden KB, Holmes-Rovner M, Llewellyn-Thomas H, Lyddiatt A, Legare F, Thomson R: **Decision aids for people facing health treatment or screening decisions.** *Cochrane Database Syst Rev* 2011:CD001431.
27. O'Connor AM, Rostom A, Fiset V, Tetroe J, Entwistle V, Llewellyn-Thomas H, Holmes-Rovner M, Barry M, Jones J: **Decision aids for patients facing health treatment or screening decisions: systematic review.** *BMJ* 1999, **319:**731-734.
28. Molenaar S, Sprangers MA, Postma-Schuit FC, Rutgers EJ, Noorlander J, Hendriks J, de Haes HC: **Feasibility and effects of decision aids.** *Med Decis Making* 2000, **20:**112-127.
29. Abhyankar P, Velikova G, Summers B, Bekker HL: **Identifying components in consent information needed to support informed decision making about trial particiaption: An interview study with women managing cancer.** *Social Science & Medicine* 2016, **161:**83-91.
30. The association for Palliative Medicine of Great Britain and Ireland Cnipcrg, Marie Curie Cancer Care, National Council for palliative Care and Palliative care Section of the Royal

For peer review only

Society of Medicine,: **Commissioning Guidance for Specialist Palliative Care: Helping to deliver commissioning objectives.** London; 2012.

31. The Department of Health: **End of Life Care Strategy – promoting high quality care for all adults at the end of life.** 2008.

32. National Institute for Health and Care Excellence: **Quality Standard for end of life care for adults.** 2013.

For peer review only
