# Supplementary material for: Developing a complex intervention to support timely engagement with palliative care for patients with advanced cancer in primary and secondary care in the UK: a study protocol
Source: BMJ Open. 2018 May 14;8(5):e022835. doi: 10.1136/bmjopen-2018-022835 (PMC5961581; doi:10.1136/bmjopen-2018-022835)
Supplement: Reviewer comments [file bmjopen-2018-022835.reviewer_comments.pdf]

PEER REVIEW HISTORY

BMJ Open publishes all reviews undertaken for accepted manuscripts. Reviewers are asked to complete a checklist review form (**http://bmjopen.bmj.com/site/about/resources/checklist.pdf**) and are provided with free text boxes to elaborate on their assessment. These free text comments are reproduced below.

ARTICLE DETAILS

**TITLE (PROVISIONAL)**	Developing a complex intervention to support timely engagement with palliative care for patients with advanced cancer in primary and secondary care in the UK: A study protocol
**AUTHORS**	Hackett, Julia; Bekker, Hilary; Bennett, Michael; Carder, Paul; Gallagher, Jean; Henry, Claire; Kite, Suzanne; Taylor, Sally; Velikova, Galina; Ziegler, Lucy

This article was not externally reviewed at BMJ Open. Protocols that have been independently assessed prior to submission to BMJ Open are usually fast-tracked to publication on the grounds that further substantial changes will not be possible. This independent assessment will usually be external, independent review for both a substantial grant award from a non-commercial or government funder and ethics approval.
